# Prosthetic Joint Infection of a Revision Knee Arthroplasty with *Candida parapsilosis*

**DOI:** 10.1155/2019/3634519

**Published:** 2019-12-16

**Authors:** Martine Christine Keuning, Aziz Al Moujahid, Wierd Pieter Zijlstra

**Affiliations:** ^1^Department of Orthopaedic Surgery, Medical Center Leeuwarden, Leeuwarden, Netherlands; ^2^Center for Infectious Diseases Friesland, Izore, Leeuwarden, Netherlands

## Abstract

We report a case of an infected total knee arthroplasty with *Candida parapsilosis*. The patient was successfully treated with a two-stage exchange arthroplasty, local antifungal treatment, and systemic antifungal treatment. This specific combination therapy to treat *C. parapsilosis* joint infection has not been previously reported.

## 1. Introduction

Prosthetic joint infections (PJIs) continue to pose challenges in total joint arthroplasty. Although PJIs are a dreaded complication and a common cause of revision surgery, treatment guidelines have made gram-positive or gram-negative PJIs controllable. A rare PJI, though increasing in prevalence, is a fungal infection. Fungal PJI represents 1% of all PJIs [1–3].

If fungal pathogens are isolated from periprosthetic tissue or joint aspirations, it can be considered a fungal PJI. Isolating the infecting organism can be a challenge with fungi, and repeated joint aspiration may be needed [4]. Management of fungal PJI, surgical as well as therapeutic, is considered more challenging due to the higher risk of persistent infection [5]. Current literature advises a two-stage exchange arthroplasty, combined with local and systemic antifungal therapy to manage fungal PJI [3–7].

Systemic as well as local antifungals can be used in medical treatment of the fungal PJI. Most frequent agents for a systemic treatment are fluconazole and amphotericin B given either orally or intravenously [4, 5]. Local antifungal agent administration can be applied by implanting an impregnated cement spacer, by placing intra-articular powder, or by daily intra-articular lavage. From these three, spacers loaded with antifungal drugs have mainly been reported [4].

PJIs with fungi are referred to as difficult to treat, and treatment has not yet been well described [1, 3, 5, 7]. Relatively few case reports and literary reviews have been published assessing a fungal PJI, mostly following different treatment regimens. Due to its rarity, no standard guidelines exist for the diagnosis and treatment of these infections. Recent proceedings of the International Consensus Meeting on Periprosthetic Joint Infection, though based on limited level of evidence, did establish recommendations regarding fungal PJI [4].

Our case describes a case of an infected total knee arthroplasty with *Candida parapsilosis*. The patient was successfully treated with a two-stage exchange arthroplasty, local antifungal treatment, and unique systemic antifungal treatment.

## 2. Case Report

A 72-year-old woman, diagnosed with rheumatoid arthritis and psoriasis, received a right total knee arthroplasty (TKA) in August 2009 for primary arthrosis with complaints of a large Baker's cyst. The procedure and follow-up went uneventfully. In May 2016, the patient returned with large swelling in the right knee cavity. A white cell scan showed signs of a prosthetic joint infection of the TKA. A DAIR (debridement, antibiotics, and implant retention) was performed on June 10 (2016) and because of persistent wound leakage again on June 27. All cultures remained sterile. No clinical improvement was followed, and therefore, the TKA was removed on July 14 (2016) and a cement spacer was placed. These cultures showed *Staphylococcus aureus* and *Staphylococcus epidermidis*, for which antibiotic treatment was started. The therapeutic regimen consisted of flucloxacillin 1200 mg daily intravenously combined with rifampicin 450 mg twice daily for 2 weeks, followed by moxifloxacin 400 mg once daily combined with rifampicin 450 mg twice daily for 6 weeks.

In February 2017, the right TKA was reimplanted (Scorpio NRG, posterior stabilized), whilst vancomycine was given. The perioperative cultures remained negative, and the vancomycine was stopped after 2 weeks and replaced with linezolid for 4 weeks. Patient follow-up in the months after the reimplantation was good, and there were no signs of infection, until January 2018, when she returned with swelling and pain in the right knee. On admission, laboratory studies revealed the following: C-reactive protein (CRP) (16 mg/ml) and erythrocyte sedimentation rate (ESR) (67 mm/h). X-ray of the knee showed a total knee prosthesis with no signs of loosening or osteolysis. A white cell scan was done which was inconclusive. A preoperative diagnostic arthrocentesis was performed. The drained synovial fluid was sent to the clinical microbiological laboratory for culture. By day 3 of incubation, the culture showed yeast using BBL CHROMagar Candida Medium (Becton Dickinson). The yeast was identified as *Candida parapsilosis* by using MALDI-TOF-MS (matrix-assisted laser desorption ionization/time-of-flight mass spectrometry). A diagnosis of chronic infection of total knee arthroplasty with *C. parapsilosis* was established. After which, the decision to undertake a two-stage revision procedure was made. The *C. parapsilosis* isolate showed susceptibility to voriconazole (minimum inhibitory concentration (MIC), 0.063 *μ*g/ml), amphotericin B (MIC, 0.125 *μ*g/ml), 5-fluorocytosine (MIC, 0.125 *μ*g/ml), fluconazole (MIC, 2 *μ*g/ml), micafungin (MIC, 0.25 *μ*g/ml), anidulafungin (MIC, 1 *μ*g/ml), and caspofungin (MIC, 1 *μ*g/ml) (Table 1). An antifungal susceptibility test was performed according to the European Committee on Antimicrobial Susceptibility Testing (EUCAST) criteria. Minimal inhibitory concentrations (MICs) were established by using a microdilution-based method, and the MICs were interpreted according to the EUCAST breakpoint (Table 1).

On March 8, 2018, the TKA was extracted. After the joint had been accessed via the preexisting incision, the patient underwent rigorous debridement of infected and devitalized tissues. The total knee arthroplasty components were removed. Once debridement was performed, the joint was extensively irrigated using iodine and 6 l of saline solution. After preparation of the joint cavity, an amphotericin B-impregnated spacer was inserted. During this procedure, 8 separate intraoperative tissue samples from various anatomical sites were collected and sent for culture. After 3 days of incubation, all eight intraoperative cultures showed *C. parapsilosis*. Blood cultures remained negative. Postoperatively, combination antifungal therapy was started. The patient was treated with intravenous voriconazole (with a loading dose of 6 mg/kg twice daily on the first day, followed by a maintenance dose of 4 mg/kg twice daily) and micafungin 200 mg once daily. Intravenous combination antifungal treatment was maintained for 4 weeks before switching to oral voriconazole monotherapy 300 mg twice daily for additional 4 weeks. Two weeks after taking the medication, the patient experienced a moderate alteration of liver enzyme levels, which was attributed to the use of micafungin. Therefore, the dose of micafungin was reduced from 200 mg to 100 mg once daily for 5 days until the enzyme levels returned to normal.

On May 18, 2018, the TKA was reimplanted. Two weeks before reimplantation, voriconazole was discontinued in order to obtain reliable tissue samples for culture. During this surgical procedure, the spacer was removed and a new semiconstrained prosthesis with a stemmed tibial component (Triathlon Total Stabilizer) was implanted. Augmentation was used for the femur (5 medial and 10 lateral) and tibia (10 medial). Also, several tissue samples were obtained preoperatively and sent for culture. Postoperatively, the patient received cefuroxime empirically and 2 weeks of combination therapy of intravenous voriconazole (with a loading dose of 6 mg/kg twice daily on the first day for 2 doses, followed by a maintenance dose of 4 mg/kg twice daily) and micafungin 200 mg once daily. After two weeks of incubation, culture of intraoperative specimen during reimplantation yielded one colony of *C. parapsilosis* in one out of seven samples with the same antibiogram as described previously; therefore, the intravenous regimen was followed up by 2 weeks of voriconazole 300 mg twice daily. Since bacterial cultures remained negative, cefuroxime was discontinued after 7 days.

Since reimplantation, the patient has been doing well and there have been no more complaints of pain and dysfunction of the knee. She reports no functional impairment. The last follow-up was June 2019. Lab results then showed CRP of 4 mg/ml, BSE of 56 mm/h (which can be related to rheumatoid arthritis), and white blood cell count of 9.7 × 10^9^.

## 3. Discussion

The treatment of fungal prosthetic joint infections remains a diagnostic and therapeutic challenge. A fungal PJI is difficult to eradicate, partly because of the considerable diagnostic delay for weeks to months due to the indolent clinical presentation. Repeated removal and reimplantation of the prosthetic joint may be necessary, sometimes resulting in a permanent removal of the prosthetic joint. Chance of failure (persistent infection) after surgical and antifungal treatment is 15-23% [5, 6, 8]. These infections often occur in immunocompromised patients (our patient has a medical history of rheumatoid arthritis) and patients who underwent previous surgeries of the affected joint (which also applies to our patient) [4–6].

We therefore considered this PJI difficult to treat. Following standard PJI guidelines and current literature on fungal PJIs [4, 5], the patient was therefore treated with a two-stage exchange arthroplasty. This treatment regimen involved removal of the implant, a rigorous surgical debridement, placement of a spacer supplemented with antifungals mixed with the bone cement, and a new combination of systemic antifungal therapy. We believe that this combination made reimplantation of the TKA thus far successful.

Due to the lack of clinical trials and the paucity of reported cases, the optimal treatment strategy for patients with fungal PJI remains a topic of discussion. The therapeutic approach is mainly based on reported experience and anecdotal evidence. Biofilm formation is considered a virulence factor of *C. parapsilosis* in PJI. To eradicate these infections, prolonged antifungal treatment with an antifungal agent that displays good biofilm activity is required. Micafungin is an example with these properties against *Candida* species.

Recent evaluation studies of susceptibility of *Candida* biofilms showed that the echinocandins, particularly micafungin, had the highest antifungal activities [9]. The use of systemic amphotericin B to treat osteomyelitis is not recommended [10]. Voriconazole achieves adequately high therapeutic concentrations in bone tissue, and micafungin is active against slow-growing organisms and biofilms. Given the mentioned pharmacokinetics and pharmacodynamics and the low MIC value of voriconazole, the combination of voriconazole and micafungin was considered an ideal therapeutic option to manage this infection.

To the best of our knowledge, this combination therapy to treat *C. parapsilosis* joint infection has not been previously reported and makes this case report unique.

## 4. Conclusion

Based on the positive therapeutic result of this case of PJI with *Candida parapsilosis*, we would advise a two-stage exchange arthroplasty combined with local antifungals mixed in bone cement and the combination of systemic voriconazole and micafungin as a new treatment option.

## Figures and Tables

**Table 1:** *Candida parapsilosis* antibiogram.

Antifungal agent	MIC (mg/l)	EUCAST guidelines		Interpretation
		Susceptible	Resistant	
Amphotericin B	**0.125**	**1**	**1**	Susceptible
Anidulafungin	**0.5**	**0.002**	**4**	Susceptible
Caspofungin	**1**	Note 1	Note 1	
Fluconazole	**2**	**2**	**4**	Susceptible
Micafungin	**0.25**	**0.002**	**2**	Susceptible
Voriconazole	**0.063**	**0.125**	**0.25**	Susceptible

Note 1: EUCAST breakpoints have not yet been established for caspofungin, due to significant interlaboratory variation in MIC ranges for caspofungin.
